# Dynamic Arterial Elastance Is Associated With the Vascular Waterfall in Patients Treated With Norepinephrine: An Observational Study

**DOI:** 10.3389/fphys.2021.583370

**Published:** 2021-05-04

**Authors:** Stéphane Bar, Maxime Nguyen, Osama Abou-Arab, Hervé Dupont, Belaid Bouhemad, Pierre-Grégoire Guinot

**Affiliations:** ^1^Department of Anaesthesiology and Critical Care, Amiens University Hospital, Amiens, France; ^2^Department of Anaesthesiology and Critical Care, Centre Hospitalier Regional Universitaire De Dijon, Dijon, France; ^3^Université Boulogne Franche Comté, LNC UMR1231, Dijon, France

**Keywords:** dynamic arterial elastance, norepinephrine, waterfall phenomenon, vascular resistance, cardiac output

## Abstract

**Introduction:** It has been suggested that dynamic arterial elastance (Ea_dyn_) can predict decreases in arterial pressure in response to changing norepinephrine levels. The objective of this study was to determine whether Ea_dyn_ is correlated with determinants of the vascular waterfall [critical closing pressure (CCP) and systemic arterial resistance (SARi)] in patients treated with norepinephrine.

**Materials and Methods:** Patients treated with norepinephrine for vasoplegia following cardiac surgery were studied. Vascular and flow parameters were recorded immediately before the norepinephrine infusion and then again once hemodynamic parameters had been stable for 15 min. The primary outcomes were Ea_dyn_ and its associations with CCP and SARi. The secondary outcomes were the associations between Ea_dyn_ and vascular/flow parameters.

**Results:** At baseline, all patients were hypotensive with Ea_dyn_ of 0.93 [0.47;1.27]. Norepinephrine increased the arterial blood pressure, cardiac index, CCP, total peripheral resistance (TPRi), arterial elastance, and ventricular elastance and decreased Ea_dyn_ [0.40 (0.30;0.60)] and SARi. Ea_dyn_ was significantly associated with arterial compliance (C_A_), CCP, and TPRi (*p* < 0.05).

**Conclusion:** In patients with vasoplegic syndrome, Ea_dyn_ was correlated with determinants of the vascular waterfall. Ea_dyn_ is an easy-to-read functional index of arterial load that can be used to assess the patient’s macro/microcirculatory status.

**Clinical Trial Registration:** ClinicalTrials.gov #NCT03478709.

## Introduction

Arterial hypotension in relation to vasoplegic syndrome is one of the most important causes of acute circulatory failure in the intensive care unit (ICU; Levin et al., 2009; Fischer and Levin, 2010). This condition is usually treated by the intravenous (IV) administration of a vasoactive agent, such as norepinephrine. The assessment of vascular tone is difficult to carry out in daily practice because the vascular parameters used (peripheral resistance and arterial compliance) did not take into account the complexity of the cardiovascular system which includes two pressure systems with a waterfall phenomenon at the microcirculatory level (Maas et al., 2012). It has been suggested that dynamic arterial elastance (Ea_dyn_) is a marker of vascular tone that can help the physician to manage the treatment of acute vasoplegic circulatory failure (Monge Garcia et al., 2011; Pinsky, 2011; Bouchacourt et al., 2013; Cecconi et al., 2015; Guinot et al., 2015). Ea_dyn_ is calculated using the ratio of respiratory pulse pressure variation (PPV) over the respiratory stroke volume variation (SVV). Indeed, it has been shown that Ea_dyn_ can predict (i) the increase in mean arterial pressure (MAP) following a fluid challenge (Monge Garcia et al., 2011) and (ii) the decrease in blood pressure following a decrease in the norepinephrine dose (Guinot et al., 2015, 2017; Nguyen et al., 2021). It is a simple marker, is easy to read, and does not require additional monitoring than that carried out routinely in intensive care (Nguyen et al., 2021). However, the physiological significance of Ea_dyn_ has never been unambiguously demonstrated. Some studies have evidenced an association between Ea_dyn_ and several components of blood pressure: MAP, effective arterial elastance (E_A_), and arterial compliance (C_A_; Guinot et al., 2017; Monge García et al., 2017; Bar et al., 2018a).

The concept of vascular waterfall introduced the existence of a pressure gradient between the critical closing pressure (CCP) at the arteriolar level and the mean systemic filling pressure (MSP) at the venular level. The CCP has been described on the microcirculatory level and corresponds to the arterial pressure below which blood flow is stopped by occlusion of the arterioles (Maas et al., 2012). The CCP and indexed systemic arterial resistance (SARi) may also be markers of the vascular tone at the junction between the microcirculatory and macrocirculatory systems. It has been shown that the changes in the vascular waterfall induced by sympathetic stimulation and arterial vasodilation are similar to those observed for Ea_dyn_ (Gaskell and Krisman, 1958; Sanders et al., 1988). The association between Ea_dyn_ and vascular waterfall components (CCP and SARi) in vasoplegic patients in the ICU has not previously been evaluated. Given that vasomotor manipulation changes CCP and SARi, we hypothesized that Ea_dyn_ indirectly reflects the status of these vascular waterfall components.

The main objective of the present study was to determine whether Ea_dyn_ is associated with the vascular waterfall components in post-operative cardiac surgery patients treated with norepinephrine. The secondary objectives were to assess the putative associations between Ea_dyn_ and several arterial load and blood flow variables.

## Materials and Methods

### Ethics

The study was approved by the local independent ethics committee (Comité de Protection des Personnes Est II, Besançon, France; reference: 17/07, June 19, 2017) and was registered at Clinicaltrials.gov (NCT03478709). The present report was drafted in line with the STARD statement (Bossuyt et al., 2015). The study complied with the tenets of the Declaration of Helsinki. All patients provided their written informed consent to participation.

### Patients

We performed a bicentric, prospective, observational study in the cardiothoracic and vascular ICU in two university hospitals. The inclusion criteria were as follows: adult patients admitted to the ICU and for whom the physician had decided, in accordance with the unit’s care protocols, to treat post-operative vasoplegic syndrome; monitoring with a central venous access and an invasive blood pressure measurement; and the presence of a sinus heart rate (HR). Vasoplegic syndrome was defined by persistent arterial hypotension despite fluid resuscitation (Abou-Arab et al., 2018, 2020). The non-inclusion criteria were the presence of a pacemaker, atrial fibrillation, ventricular tachycardia, or ventricular fibrillation; age under 18 years; prior use of vasoactive/inotropic support; or right heart failure.

### Hemodynamic Measurements

We measured the systolic blood pressure (SAP), diastolic blood pressure (DAP), and MAP using an invasive arterial catheter.

Echocardiography (using a CX50 ultrasound system and S5-1 Sector Array Ultrasound Probe Transducer, Philips Medical Systems®, Suresnes, France) was performed by a physician who was blinded to the study outcomes. The left ventricular ejection fraction (LVEF) was measured using Simpson’s biplane method with a four-chamber view. The indexed stroke volume (SVi; ml) was calculated by multiplying the left ventricular outflow tract’s (LVOT’s) velocity time integral (VTI) by the aortic area and dividing by body surface area (BSA). The cardiac index (CI) was calculated as SVi × HR.

The respiratory SVV was calculated as (SVmax − SVmin)/[(SVmax + SVmin)/2] × 100.

Mean echocardiographic parameters were calculated from five individual beat measurements (regardless of the respiratory cycle) and analyzed retrospectively by a senior cardiologist with echocardiography certification.

#### MSP and CCP

The MSP and CCP were measured using the inspiratory breath-hold maneuver described by Maas et al. (2012). The measured venous and arterial pressures were plotted against cardiac output (CO), and a linear regression line was fitted to the data points. The MSP was determined by extrapolation of the central venous pressure (CVP) to zero flow on the venous return curve. The CCP was determined by extrapolation of the arterial pressure to zero flow on the ventricular output curve. Indexed SARi (mmHg ml^−1^·m^−2^) was calculated as (MAP − CCP)/CI. Indexed systemic venous resistance (VRi, mmHg ml^−1^·m^−2^) was calculated as (MSP − CVP)/CI.

#### Left Ventricular End-Systolic Elastance, Arterial Elastance, and Ventricular-Arterial Coupling

Left ventricular end-systolic elastance (E_V_) was estimated at the bedside by using the equation E_V_ = MAP/(ESVi-4; mmHg ml^−1^·m^−2^; Tanaka et al., 1998). This measure non-invasively used echocardiographic measurements of end-systolic volume index (ESVi). This method was used in cardiologic studies to assess the effect of vasoactive medication on ventriculo-arterial coupling (Asanoi et al., 1989; Shmuel et al., 2012).

E_A_ is an index that integrates the main components of arterial load, i.e., total peripheral resistance (TPRi), total net C_A_, characteristic impedance, and systolic and diastolic time intervals (Tanaka et al., 1998; Ky et al., 2013). E_A_ was estimated by using the equation E_A_ = MAP/SVi (mmHg ml^−1^·m^−2^). This ratio has been shown to be a robust E_A_ surrogate over a wide range of hemodynamic conditions and is interchangeable in any peripheral artery (Monge Garcia et al., 2018).

Ventricular-arterial coupling was calculated as E_A_/E_V_ ratio_._

TPRi was calculated as (MAP − CVP)/CI (mmHg ml^−1^·m^−2^), and C_A_ (ml mmHg^−1^·m^−2^) was calculated as SVi/arterial pulse pressure (PP).

Ea_dyn_ was calculated as the ratio between the PPV and the SVV (Guinot et al., 2017).

### Study Protocol

The indication for treatment with norepinephrine was left to the discretion of the physician in charge of the patient. The following clinical parameters were recorded: age, gender, weight, height, body mass index, Euroscore 2, medical history, operating time, and cardiopulmonary bypass duration. After an equilibration period, baseline measurements of HR, SAP, MAP, DAP, MSP, TPRi, SVi, CI, SVV, PPV, and vascular determinants of waterfall were recorded. A second (post-norepinephrine) set of measurements was recorded after 15 min of hemodynamic stability (defined as a change in MAP of less than 10%). All patients had been sedated by continuous infusion of propofol and were fully adapted to mechanical ventilation. All patients were mechanically ventilated in pressure-controlled mode with a positive end-expiratory pressure (PEEP) of 5 cmH_2_O. Ventilator settings and the propofol infusion rate were the same for the two sets of measurements.

### Statistical Methods

We calculated that a sample size of 14 patients would allow to demonstrate a correlation between Ea_dyn_ and CCP higher than 0.5, using Spearman’s correlation coefficient with a risk alpha of 5% and a power of 80%. The distribution of the variables was assessed using the D’Agostino-Pearson test. Data are expressed as proportion (%), mean (standard deviation), or median [IQR] (interquartile range) as appropriate. Paired data were compared using Wilcoxon signed rank test. Linear correlations were tested using Spearman’s rank method. Associations between arterial load and blood flow parameters measurement considering both time points were assessed using mixed linear modeling. Only bivariate models were carried out. Ea_dyn_ was the dependent variable; each hemodynamic variable was used as fixed effect, and individual was used as random intercept. The normality of the distribution of random effects and of the model residual were graphically checked. Because this analysis was exploratory, slight disparities between those distributions and normality were tolerated. The threshold for statistical significance was set to 0.05. Statistical analysis was performed using RStudio Version 1.1.447-2009-2018 RStudio, Inc., and SPSS® software (version 24, IBM, New York, NY, United States).

## Results

Twenty patients were included during the study period. Three were excluded because of incomplete data, and so 17 were included in the final analysis. Most of the study participants were male (80%). The median age was 68 [50;71], and the median Euroscore 2 was 2.3 [1.5;4.3] (Table 1). The median dose of norepinephrine was 0.08 gamma kg^−1^·min^−1^ [0.06;0.11]. None of the patients had been treated with inotropic agents or vasopressors before enrolment in the study.

**Table 1:** Demographic data.

Variables	(*N* = 20)
Age (years)	68 [50–71]
Male (%)	16 (80%)
Weight (kg)	85 ± 18
Height (cm)	175 ± 9
BMI (kg·m^−2^)	27.9 ± 4.8
Euroscore 2 (%)	2.3 [1.5–4.3]
Medical history, *n* (%)	
High blood pressure	13 (65%)
Diabetes	0 (0%)
Coronary artery disease	5 (25%)
Smoking	11 (55%)
Dyslipidemia	6 (30%)
Surgery, *n* (%)	
Valve surgery	10 (50%)
Coronary artery bypass graft	7 (35%)
Both of the above procedures	3 (15%)
Cardiopulmonary bypass duration (min)	117 [91–158]
Length of stay in the ICU (days)	3 [2–5]
Length of stay in hospital (days)	11 ± 3

The data were quoted as the number (%), mean ± standard deviation SD, or the median [interquartile range, IQR], as appropriate. BMI, body mass index; ICU, intensive care unit.

At baseline, patients had arterial hypotension, with low CCP and CI values, and high SARi, SVV, PPV, and Ea_dyn_ values (Table 2). Infusion of norepinephrine significantly increased SAP, DAP, MAP, PP, CVP, CCP, TPRi, VRi, SVV, SVi, CI, E_A_, and E_V_ and significantly decreased Ea_dyn_, PPV, C_A_, and SARi (Table 2). These changes did not differ between non-smoker and smoker patients (*p* = 0.45).

**Table 2:** Comparison of hemodynamic parameters at baseline and after the norepinephrine injection.

Hemodynamic variables	Baseline	Post-injection	*p*
HR (bpm)	75.5 [63.0;91.2]	79.0 [64.2;91.8]	0.491
SAP (mmHg)	89 [84; 94]	122 [114;130]	**0.001**
DAP (mmHg)	46.5 [41.0;50.2]	62.5 [55.5;67.2]	**0.001**
MAP (mmHg)	60.5 [56.0;66.0]	83.0 [76.5;89.0]	**0.001**
PP (mmHg)	41.5 [35.0;46.5]	57.5 [49.8;68.2]	**<0.001**
CVP (mmHg)	7.00 [5.50;9.00]	8.50 [5.75;9.25]	**<0.001**
SVV (%)	17.2 [15.0;23.7]	25.0 [21.9;33.3]	**0.002**
PPV (%)	14.5 [10.5;18.2]	9.50 [7.00;14.5]	**0.027**
Ea_dyn_	0.93 [0.47;1.27]	0.40 [0.30;0.60]	**0.042**
MSP (mmHg)	23.0 [18.5;26.0]	19.0 [14.8;24.0]	0.149
VRi (mmHg ml^−1^·m^−2^)	2.79 [2.63;2.98]	3.06 [2.76;3.26]	**0.005**
CCP (mmHg)	26.1 [19.4;29.1]	54.8 [39.8;70.3]	**0.001**
SARi (mmHg ml^−1^·m^−2^)	18.4 [14.5;25.8]	11.4 [6.06;18.5]	**0.002**
CCP-MSP (mmHg)	5.74 [−3.8;11.1]	42.0 [17.1;48.0]	**0.001**
SVi (ml·m^−2^)	23.0 [17.5;28.8]	28.0 [19.0;36.0]	**0.034**
CI (ml min^−1^·m^−2^)	1.66 [1.56;2.09]	1.96 [1.82;2.32]	**0.010**
E_A_ (mmHg ml^−1^·m^−2^)	1.66 [1.41;2.31]	1.75 [1.50;3.25]	**0.003**
Total C_A_ (ml mmHg^−1^·m^−2^)	1.27 [1.01;1.61]	0.95 [0.78;1.22]	**0.016**
TPRi (mmHg ml^−1^·m^−2^)	29.0 [24.5;39.0]	36.0 [29.2;43.0]	**0.004**
Ventricular elastance (E_V_; mmHg ml^−1^·m^−2^)	1.2 [0.9;1.9]	1.74 [1.1;2.4]	**0.002**
E_A_/E_V_ ratio (units)	1.4 [1.1;1.9]	1.3 [1;1.9]	0.215

Values are expressed as the mean ± SD or the median [IQR]. C_A_, arterial compliance; CI, cardiac index; CVP, central venous pressure; DAP, diastolic arterial pressure; E_A_, arterial elastance; Ea_dyn_, dynamic arterial elastance; E_V_, ventricular elastance; PPV, pulse pressure variation; HR, heart rate; MAP, mean arterial pressure; CCP, critical closing pressure; PP, pulse pressure; TPRi, indexed total peripheral resistance; SAP, systolic arterial pressure; SARi, indexed systemic arterial resistance; SVi, indexed stroke volume; SVV, stroke volume variation; VRi, indexed systemic venous resistance. The value of *p* refers to the within-group (before/after) comparison. Bold values are values with p < 0.05.

**Figure 1:**
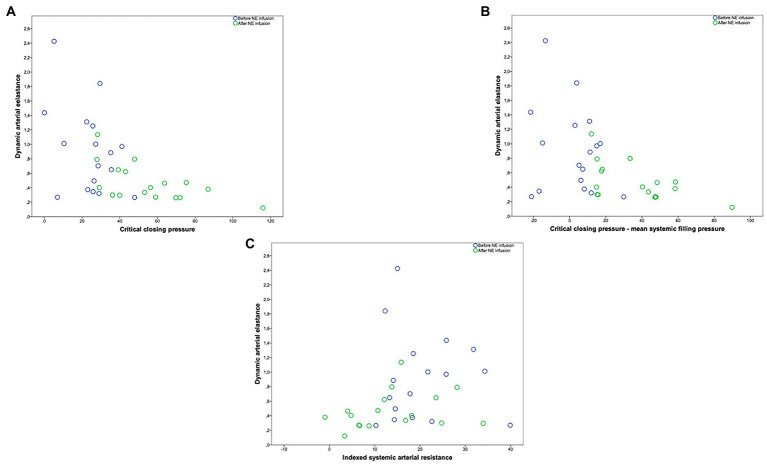
**(A)** Relationship between dynamic arterial elastance and critical closing pressure (mmHg). **(B)** Relationship between dynamic arterial elastance and critical closing pressure minus mean systemic filling pressure (mmHg). **(C)** Relationship between dynamic arterial elastance and indexed systemic arterial resistance (mmHg ml^−1^·m^−2^). Circle color refers to before (blue circle)/after norepinephrine (NE) infusion (green circle).

**Figure 2:**
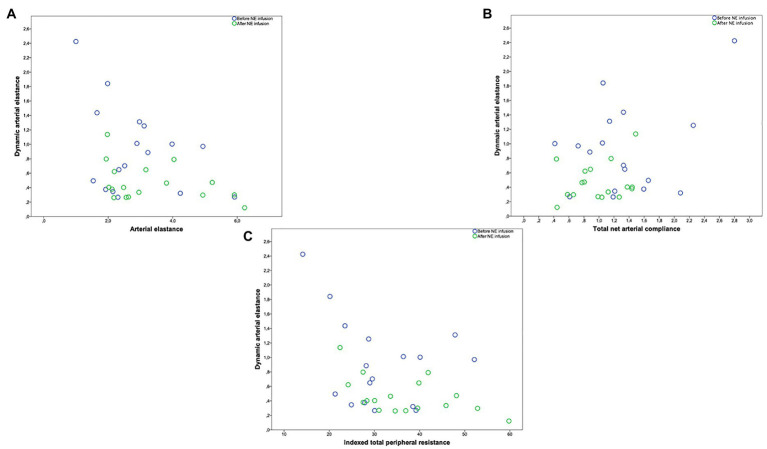
**(A)** Relationship between dynamic arterial elastance and arterial elastance (mmHg ml^−1^·m^−2^). **(B)** Relationship between dynamic arterial elastance and total net arterial compliance (ml mmHg^−1^·m^−2^). **(C)** Relationship between dynamic arterial elastance and indexed total peripheral resistance (mmHg ml^−1^·m^−2^). Circle color refers to before (blue circle)/after norepinephrine (NE) infusion (green circle).

In the overall study population, Ea_dyn_ was significantly and negatively correlated with CCP (*r* = −0.557, *p* = 0.0017), CCP-MSP (*r* = −0.515, *p* = 0.003), SARi (*r* = −0.487, *p* = 0.003), SAP (*r* = −0.402, *p* = 0.004), DAP (*r* = −0.484, *p* = 0.005), MAP (*r* = −0.498, *p* = 0.004), E_A_ (*r* = −0.347, *p* = 0.05), C_A_ (*r* = −0.406, *p* = 0.021), and TPRi (*r* = −0.396, *p* = 0.025; Figures 1, 2). Ea_dyn_ was not correlated with LVEF (*r* = 0.012, *p* = 0.945).

The linear mixed model used to analyze the contribution of arterial and blood flow parameters to Ea_dyn_ demonstrated that Ea_dyn_ was significantly associated with C_A_, blood pressure, CCP, and TPRi (Table 3).

**Table 3:** Estimated values of fixed effects (arterial load and cardiac parameters) on Ea_dyn_ according to a linear mixed-effects model analysis.

Covariates	Ea_dyn_	*p*
*Arterial covariates*		
SAP (mmHg)	−0.02 (0.01)	**0.040**
DAP (mmHg)	−0.03 (0.01)	**0.040**
MAP (mmHg)	−0.03 (0.01)	**0.030**
PP (mmHg)	0.19 (0.04)	0.190
CCP (mmHg)	−0.55 (0.02)	**0.020**
SARi (mmHg ml^−1^·m^−2^)	−0.11 (0.04)	0.730
E_A_ (mmHg ml^−1^·m^−2^)	−0.43 (0.22)	0.070
C_A_ (ml mmHg^−1^·m^−2^)	1.25 (0.33)	**0.010**
TPR*i* (mmHg ml^−1^·m^−2^)	−0.05 (0.02)	**0.020**
Norepinephrine dose (g kg^−1^ min^−1^)	−5.9 (3.3)	0.090
*Cardiac covariates*		
HR (beats min^−1^)	−0.01 (0.01)	0.230
LVEF (%)	0.016 (0.011)	0.670
E_V_ (mmHg ml^−1^·m^−2^)	−0.17 (0.22)	0.330
E_A_/E_V_ (units)	−0.10 (0.038)	0.610
CI (l min^−1^·m^−2^)	0.89 (0.36)	**0.020**

Data are presented as an estimated value (SE). C_A_, arterial compliance; CI, cardiac index; CVP, central venous pressure; DAP, diastolic arterial pressure; E_A_, arterial elastance; Ea_dyn_, dynamic arterial elastance; E_V_, ventricular elastance; NE, norepinephrine; HR, heart rate; LVEF, left ventricular ejection fraction; MAP, mean arterial pressure; MSP, mean systemic pressure; CCP, critical closing pressure; PP, pulse pressure; TPRi, indexed total peripheral resistance; SAP, systolic arterial pressure; SARi, indexed systemic arterial resistance. Bold values are values with p < 0.05.

## Discussion

The present study’s main finding was the association between Ea_dyn_ and determinants of the vascular waterfall (CCP and SARi). Furthermore, we confirmed that Ea_dyn_ is associated with several components of arterial load (C_A_ and TPRi) and blood pressure (MAP and CCP).

According to the concept of macrocirculatory-microcirculatory coherence, any change in the macrocirculation will affect the microcirculation (Bennett et al., 2018). Hence, resuscitation procedures intended to correct macrocirculatory hemodynamic variables will also modify regional and microcirculatory perfusion and oxygen delivery to the tissues (Ince, 2015). The CCP was described at the microcirculatory level and represents the arterial pressure below which blood flow is stopped by occlusion of the arterioles (Versluis et al., 2001; Maas et al., 2012). The vascular waterfall is thought to maintain tissue perfusion when blood flow is abnormally low. The CCP values observed in the present study were similar to those published in the literature (Jellinek et al., 2000; Schipke et al., 2003; Kottenberg-Assenmacher et al., 2009). As expected, norepinephrine increased CCP, the vascular waterfall (i.e., the CCP-MSP difference), and thus the level of tissue perfusion.

When arterial hypotension occurs, the main adaptive mechanism is the maintenance of blood flow to the tissues. In this respect, it has been suggested that several adaptive hemodynamic factors are activated so as to maintain the blood flow redistribution (De Hert, 2012). The blood pressure increase with norepinephrine treatment might restore the blood pressure and thus diminish the contribution of these hemodynamic factors to changes in tissue perfusion. Hence, we observed a decrease in SARi, which might be due to the redistribution of blood flow to non-perfused capillaries (microvascular recruitment) with norepinephrine, and a decrease in the adaptive phenomena described above (Georger et al., 2010; De Backer et al., 2014).

**Figure 3:**
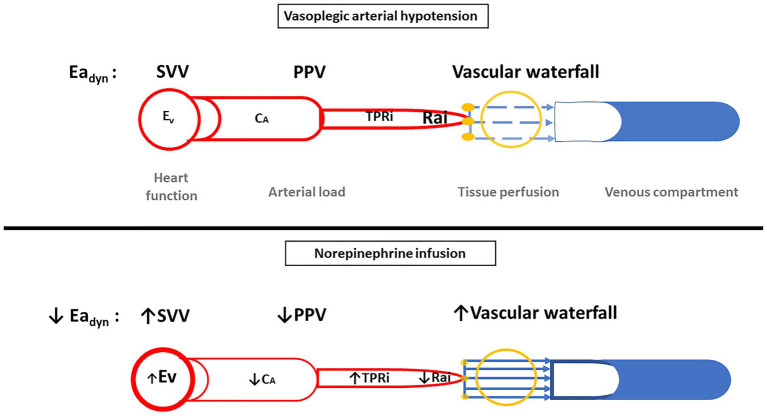
Illustration of the evolution of parameters in cases of vasoplegic arterial hypotension and in case of norepinephrine infusion. C_A_, arterial compliance; CCP, critical closing pressure; E_A_, arterial elastance; Ea_dyn_, dynamic arterial elastance; E_V_, ventricular elastance; PPV, pulse pressure variation; TPRi, indexed total peripheral resistance; Rai, resistance arterial indexed; SVV, stroke volume variation.

The changes in Ea_dyn_ after norepinephrine infusion contrast with those observed upon norepinephrine weaning in the patients with sepsis or after cardiac surgery (Guinot et al., 2017). Likewise, studies of Ea_dyn_ in the operating theater and in the ICU have given conflicting results (Guinot et al., 2015; Lanchon et al., 2017; Bar et al., 2018b). These disparities may be due to several factors, e.g., the non-linearity of the blood pressure-volume relationship (Sanders et al., 1988), the cardiovascular effects of norepinephrine (as an alpha and beta-agonist; Guinot et al., 2018) and the underlying disease (vasoplegia is associated with altered autonomic function; Bernardelli et al., 2016), and thus changes in the interactions between the arterial load and blood pressure. Hence, Ea_dyn_ decreased with norepinephrine infusion probably because of a decrease in C_A_, an increase in left ventricular inotropy (E_V_), and a decrease in arteriolar vasomotor tone (SARi; Figure 3).

As demonstrated by the literature data, C_A_ is probably the main contributor to Ea_dyn_ (Monge García et al., 2017; Bar et al., 2018a). It has been shown that norepinephrine infusion can restore C_A_ (Nguyen et al., 2018). Fluid expansion does not change C_A_ in patients treated with norepinephrine but decreases C_A_ in patients not treated with norepinephrine. Likewise, norepinephrine weaning is not associated with a change of C_A_ in non-pressure-responder patients (Bar et al., 2018b). Thus, in norepinephrine-treated patients, changes in the arterial load may mainly be driven by the resistive component (i.e., SARi and TPRi; Bernardelli et al., 2016; Nguyen et al., 2018). One can suppose that in patients treated with vasopressor, Ea_dyn_ mainly reflects vascular resistance because C_A_ is fixed. This hypothesis may also explain the ability of Ea_dyn_ (when measured with an uncalibrated CO device) to predict arterial pressure changes (Bar et al., 2018b). The arterial load and Ea_dyn_ may vary in a complex manner as a function of the therapeutic intervention while decreasing Ea_dyn_’s ability to predict blood pressure changes. The main negative studies concerning Ea_dyn_ were performed in the operating theater, and the positive studies were performed in the ICU (i.e., where most of the patients had received vasopressors; Vos et al., 2013; Lanchon et al., 2017; Guarracino et al., 2019). In practice, Ea_dyn_ could be considered as an easy-to-read indicator that can help the physician to choose the optimal hemodynamic strategy at the bedside. This index could be used to understand the interaction between cardiac function and arterial load, the effects of hemodynamic treatment on components of arterial load, coherence between macrocirculation and microcirculation (i.e., the vascular waterfall’s status), and thus to determine which treatments could be used or withdrawn. Guarracino et al. (2019) have demonstrated that Ea_dyn_ predicts changes in MAP upon fluid expansion or vasopressor administration. In contrast to the complex procedures required to measure the vascular waterfall and arterial load variables, Ea_dyn_ is easy to measure with hemodynamic devices – even an uncalibrated pulse contour analysis device (Bar et al., 2018b).

The present study had several limitations. Even though we predetermined the sample size, the present results included a small number of patients and will require external validation in another cohort. Our results were obtained in a group of patients with arterial hypotension treated with norepinephrine and must now be confirmed in patients treated with fluid expansion or inotropes. We did not include a control group, i.e., using IV fluids because fluid infusion may be unable to treat vasoplegia, and it seems difficult to compare the effects of fluid on vasomotor tone and those of norepinephrine in two populations who do not suffer the same hemodynamic underlying disease. To avoid mathematical coupling, we specifically chose to calculate Ea_dyn_ from two different signals. The arterial load assessment was based on a two-element Windkessel model and integrative simplification (Westerhof et al., 2009). More precise models (include arterial impedance and wave reflection) have since been developed. However, the performance of these measurements at the bedside would be challenging. The E_A_ calculated from MAP was demonstrated to be a robust surrogate over a wide range of hemodynamic conditions and can be applied to any peripheral artery (Monge Garcia et al., 2018). The method that we used to calculate E_V_ and E_A_ can be criticized because we did not use a high-fidelity catheter for ventricular pressure measurement; however, our approach has been studied previously (Huette et al., 2020; Nguyen et al., 2020). Likewise, we used a standardized inspiratory breath-hold maneuver. This method has been validated in ICU patients, and the values obtained in the present study were similar to those determined with other methods (Maas et al., 2012). Finally, there was heterogeneity in the study participants because their cause for surgery was not the same, but all patients suffer the same hemodynamic disease, and the inclusion criteria allow to homogenize the studied population.

In hypotensive patients treated with norepinephrine, Ea_dyn_ may indirectly reflect the vascular waterfall phenomenon and changes in one or both of its determinants (CCP and SARi) following treatment. Hence, one can consider that Ea_dyn_ reflects vasomotor tone in hypotensive patients treated with norepinephrine. In view of our present results and the literature data, one can further consider that Ea_dyn_ indirectly reflects macro-microcirculatory coherence during acute circulatory failure.

## Data Availability Statement

The raw data supporting the conclusions of this article will be made available by the authors, without undue reservation.

## Ethics Statement

The studies involving human participants were reviewed and approved by Comité de Protection des Personnes Est II, Besançon, France; reference: 17/07, June 19th, 2017. The patients/participants provided their written informed consent to participate in this study.

## Author Contributions

SB, MN, P-GG, OA-A, and BB designed the study and interpreted the data. SB and P-GG analyzed the data. SB, P-GG, and HD drafted the manuscript for important intellectual content. All authors contributed to the article and approved the submitted version.

### Conflict of Interest

The authors declare that the research was conducted in the absence of any commercial or financial relationships that could be construed as a potential conflict of interest.

## Figures and Tables

**Table:** 

Term	Definition
C_A_	Arterial compliance
CCP	Critical closing pressure
CI	Cardiac index
CO	Cardiac output
CVP	Central venous pressure
DAP	Diastolic arterial pressure
E_A_	Arterial elastance
Ea_dyn_	Dynamic arterial elastance
E_V_	Ventricular elastance
HR	Heart rate
LVEF	Left ventricular ejection fraction
MAP	Mean arterial pressure
MSP	Mean systemic pressure
PP	Pulse pressure
PPV	Pulse pressure variation
TPRi	Indexed total peripheral resistance
SAP	Systolic arterial pressure
SARi	Indexed systemic arterial resistance
SVi	Indexed stroke volume
SVV	Stroke volume variation
VRi	Indexed systemic venous resistance
